# Suppression of Th17 Cell Response in the Alleviation of Dextran Sulfate Sodium-Induced Colitis by *Ganoderma lucidum* Polysaccharides

**DOI:** 10.1155/2018/2906494

**Published:** 2018-05-20

**Authors:** Bing Wei, Ran Zhang, Jingbo Zhai, Junfeng Zhu, Fangli Yang, Dan Yue, Xiaoyi Liu, Changlong Lu, Xun Sun

**Affiliations:** Department of Immunology, China Medical University, Shenyang, China

## Abstract

**Background:**

*Ganoderma lucidum* polysaccharides (GLP) has anti-inflammatory and immunomodulatory effects. Dysregulated immune responses are involved in the pathogenesis of dextran sulfate sodium (DSS)-induced colitis. The aim of this study was to assess the therapeutic potential of GLP to alleviate DSS-induced colitis.

**Methods:**

The mice were administered with GLP by intragastric gavage daily for two weeks prior to the DSS treatment. Mice were orally administered with 2.5% DSS dissolved in drinking water with GLP or water treatment for 6 days. The mice were killed on day 7 after induction of colitis. Survival rates, body weight loss, colon lengths, histological changes, and disease activity index scores (DAI) were evaluated.

**Results:**

GLP significantly improved survival rates, colon length shortening, body weight loss, histopathological score, and DAI scores in mice with DSS-induced colitis. GLP markedly suppressed the secretions of TNF-*α*, IL-1*β*, IL-6, IL-17A, and IL-4 and significantly affected populations of Th17 cells, B cells, NK cells, and NKT cells in the lamina propria lymphocytes.

**Conclusions:**

GLP prevented inflammation, maintained intestinal homeostasis, and regulated the intestinal immunological barrier functions in mice with DSS-induced colitis.

## 1. Introduction

Inflammatory bowel disease (IBD) consists of two major phenotypes, ulcerative colitis (UC) and Crohn's disease (CD). IBD is a chronic disorder caused by dysregulated immune responses to intestinal microorganisms that occurs within all or parts of the intestinal tract. The prevalence of IBD continues to increase steadily in the Western countries, and even the newly industrialized countries have a rapidly increasing incidence [1]. Although the complex etiology of both CD and UC has not been definitively elucidated, imbalanced cytokine production and T cell dysfunction are responsible for the pathogenesis of IBD [2, 3]. Particularly, increased secretion of proinflammatory cytokines is considered as the key factor in the pathophysiology of IBD. In the colonic mucosa of UC patients, proinflammatory cytokines, such as tumor necrosis factor-*α* (TNF-*α*), interleukin-1*β* (IL-1*β*), and interleukin-6 (IL-6), are increased [4–6]. Furthermore, complements play a prominent role in IBD pathogenesis. Increased expression of the complement system gene has been found in IBD patients [7, 8]. Besides, L-selectin is closely associated with colitis [9, 10]. In the experimental studies, dextran sulfate sodium (DSS)-induced acute or chronic colitis is a standard model that is widely used to test the efficacy of therapeutic approaches for IBD [11, 12]. Th1 and Th2 cytokines, such as interferon-*γ* (IFN-*γ*), interleukin-4 (IL-4), and interleukin-5 (IL-5), are closely associated with the pathogenesis of colitis induced by DSS [13]. Recently, it has been reported that Th17 cells, which mainly produce IL-17A, are also involved in the progression of DSS-induced colitis [14–16].

*Ganoderma lucidum*, which is also called lingzhi in Chinese and reishi in Japanese, is a well-known traditional Chinese medicinal mushroom. It has been widely used for the prevention and treatment of various diseases as an immunomodulating agent, including gastric cancer [17], hypertension [18], arthritis [19], chronic hepatitis [20], diabetes [21], asthma [22], nephritis [23], arteriosclerosis [24], and immunological disorders [25]. Interestingly, it has been reported that *G. lucidum* grown on germinated brown rice (GLBR) attenuated colitis by DSS induction via inhibition of MAPK phosphorylation and NF-*κ*B activation [26]. Several classes of bioactive substances have been isolated and identified from *G. lucidum*, which include polysaccharides, triterpenoids, nucleosides, sterols, and alkaloids. Notably, *Ganoderma lucidum* polysaccharide (GLP) is the major biologically active component in *G. lucidum* [27], having anti-inflammatory and immunomodulatory effects. GLP can affect immune cells and immune-related cells including T lymphocytes [28], B lymphocytes [29], macrophages [30], dendritic cells [31], and natural killer (NK) cells [32]. Recent results indicated that GLP suppresses not only the complement- and cytokine-mediated inflammation but also the L-selectin-mediated inflammation both in vitro and in vivo [33]. Polysaccharides from the mycelia of *G. lucidum* could improve the intestinal mechanical barrier function and regulate the intestinal immunological barrier functions in the ileum [34]. Furthermore, the LZ-8 protein of *G. lucidum* alleviated acute intestinal inflammation in mice by inducing the expansion of Foxp3^+^ regulatory T (Treg) cells [35]. Polysaccharides from *Ganoderma lucidum* mycelia (MAK) had preventive effects against the development of chemical carcinogen-induced aberrant crypt foci (ACF), colon adenoma, colon adenocarcinoma, and pulmonary adenocarcinoma in rats [36]. However, there is no direct study regarding the efficacy of GLP on colitis and the mechanism of immune regulation.

In the present study, we investigated the protective role and the immunomodulatory effects of GLP on DSS-induced colitis in mice and explore the underlying mechanisms of these effects.

## 2. Method and Material

### 2.1. Chemicals and Reagents

Fc*γ* receptor-blocking mAb (CD16/32; 2.4G2), anti-Gr-1 (RB6-8C5), anti-CD3*ε* (145-2C11), anti-CD4 (RM4-5), anti-CD44 (IM7), anti-IFN-*γ* (XMG1.2), anti-IL-17A (TC11-18H10.1), purified anti-CD3, and anti-CD28 were purchased from BD Biosciences (San Diego, CA, USA). Anti-F4/80 (BM8), anti-CD25 (PC61), anti-B220 (RA3-6B2), anti-TCR*β* (H57-597), anti-TCR*γδ* (GL3), and anti-NK1.1 (PK136) were purchased from BioLegend (San Diego, CA, USA). GLP was obtained from Johncan Mushroom (Hangzhou, China). The polysaccharide was extracted and isolated according to the previous report [37]. Briefly, the *G. lucidum* mycelia were broken by microwave, defatted with alcohol, and extracted with 10 volume of double-distilled water for 4 h at 95–100°C. The solution was centrifuged to remove the insoluble materials. The supernatant was collected and evaporated under reduced pressure and precipitated upon addition of 3 volume of anhydrous ethanol. The resulting precipitates were collected, dissolved in distilled water, and treated with Sevag reagent [38] to remove protein and then dialyzed against distilled water for 48 h at 4°C to remove the low-molecular-weight materials. The solution was again evaporated and precipitated with an equal volume of cold anhydrous ethanol at 4°C overnight. The precipitates were collected by centrifugation and then lyophilized to obtain GLP. The content of polysaccharide in GLP determined using the phenol-sulfuric acid method [39] was 95%.

### 2.2. Animal and Grouping

Male C57BL/6 mice aged 6–8 weeks weighing 20 ± 1 g were purchased from Beijing HFK Bioscience Company (Beijing, China). The mice were housed in individually ventilated cages at a temperature of 20 ± 1°C and light-controlled cycle (12 hours) with free access to standard laboratory chow and tap water under a specific pathogen-free (SPF) environment. After one week, the mice were used in the study experiment. This study was approved by the Committee of Ethics on Animal Experiment of China Medical University. Experiments were carried out in accordance with the Guidelines of Animal Experiments.

The mice were randomly distributed into two groups containing ten animals each. The mice were administered with GLP (100 mg/kg body weight; dissolved in 0.2 mL water) by intragastric gavage every day for two weeks prior to the DSS treatment. Water (0.2 mL) was administered as a control. Mice were orally administered with 2.5% DSS (MW 36,000–50,000, MP Biomedicals, USA) dissolved in drinking water with GLP or water treatment for 6 days. The mice were killed on day 7 after induction of colitis. The colon tissue was removed and cut into pieces for histological analyses, cell culture, flow cytometry, and ELISA.

### 2.3. Evaluation of Colitis Progression

Body weights were recorded daily. Severity of colitis was assessed by the disease activity index (DAI) based on weight loss, trait of stool, and stool bleeding or hematochezia according to the classic scoring system by Cooper et al. [40]. The scoring process was given as follows: body weight loss (0, none; 1, 1%–5%; 2, 5%–10%; 3, 10%- 20%; 4, >20%), stool consistency (0, normal; 2, loose stool; 4, diarrhea), and stool blood (0, negative; 2, fecal occult blood test positive; 4, gross bleeding).

### 2.4. Histology Assessment of Colitis

The middle parts of the colon were removed and fixed with 4% paraformaldehyde and embedded in paraffin. Five mm tissue sections were stained with hematoxylin and eosin (HE). Histology was scored as follows: epithelium (E), 0 = normal morphology, 1 = loss of goblet cells, 2 = loss of goblet cells in large areas, 3 = loss of crypts, and 4 = loss of crypts in large areas; and infiltration (I), 0 = no infiltrate, 1 = infiltrate around the crypt basis, 2 = infiltrate reaching the L. muscularis mucosae, 3 = extensive infiltration reaching the L. muscularis mucosae and thickening of the mucosa with abundant edema, and 4 = infiltration of the L. submucosa. The total histological score was calculated as E + I [41].

### 2.5. Cell Preparation

Lamina propria (LP) cells in the colon were isolated by modifying the method described previously [42]. In brief, colon was cut into 1–2 mm pieces. The pieces were stirred in PBS containing 3 mM EDTA for 15 min twice and in RPMI 1640 (HyClone) containing 1 mM EGTA for 20 min twice at 37°C to eliminate the epithelium and then stirred at 37°C in RPMI 1640 containing 20% fetal bovine serum, 1 mM EGTA, and 1.5 mM MgCl_2_ for 15 min twice. The pieces were collected and stirred at 37°C in RPMI 1640 containing 20% FBS, 100 U/mL collagenase (Sigma-Aldrich Corp, USA), and 5 U/mL DNase 1 (Sigma-Aldrich Corp, USA) for 90 min. Halfway through the incubation and at the end of the incubation, the suspension was dissociated by multiple aspirations for 2 min through a syringe. The suspensions were centrifuged, and then the pellets were washed. Lamina propria lymphocytes (LPL) were purified from the LP cell preparations through a 45–66.6% discontinuous Percoll (Solarbio) gradient by centrifugation at 2500 rpm for 20 min at 25°C. The number of viable LPLs was counted after staining with trypan blue.

### 2.6. Flow Cytometry

Briefly, 1 × 10^6^ cells isolated from the colon of each mouse were incubated with an Fc*γ*R-blocking mAb and stained with mAbs against Gr-1, F4/80, CD3, B220, *αβ*TCR, *γδ*TCR, NK1.1, CD4, CD44, and CD25 for 30 min at 4°C. For intracellular cytokine staining, LPLs were stimulated with ionomycin (1 *μ*g/mL, Sigma-Aldrich) and PMA (25 ng/mL, Sigma-Aldrich) for 5 h at 37°C with Brefeldin A (10 mg/mL, Sigma-Aldrich) added after 1 h. These cells were harvested, washed, and stained with mAbs against IFN-*γ* and IL-17A for 30 min at 4°C. The cells were analyzed by intracellular cytokine FACS using a Cytofix/Cytoperm Kit Plus (BD Biosciences, San Jose, CA, USA) according to the manufacturer's instructions.

### 2.7. Cytokine Enzyme-Linked Immunosorbent Assay (ELISA)

Supernatants of cell cultures were collected after centrifugation at 1000 rpm for 10 min, and cytokine concentrations were measured using mouse immunoassay kits (R&D Systems Inc., Minneapolis, MN, United States) according to the manufacturer's protocol. The levels of TNF-*α*, IL-6, IL-1*β*, and IL-23 were measured in the supernatants without anti-CD28 (1 *μ*g/mL)/anti-CD3 (10 *μ*g/mL) mAbs stimulations. The levels of IFN-*γ*, IL-17A, IL-4, and IL-10 were measured in the culture supernatants with or without anti-CD28 (1 *μ*g/mL)/anti-CD3 (10 *μ*g/mL) mAbs stimulations for 48 h.

### 2.8. Real-Time Quantitative Polymerase Chain Reaction (RT PCR)

Total RNA was extracted from colon tissue using the RNAiso Plus (Takara, Dalian, China), according to the manufacturer's protocol. RNA was reverse transcribed to cDNA using PrimeScript™ RT Reagent Kit with gDNA Eraser (Perfect Real Time, Takara). The PCR mixture was prepared using SYBR® Premix Ex Taq™ (Tli RNase H Plus, Takara) and one of the primers listed in Table 1. The amplification protocol consisted of the following steps: 95°C for 30 s and 40 cycles of 95°C for 15 s and 60°C for 34 s on an ABI PRISM 7500 Sequence Detection System (Applied Biosystems, Foster City, CA). The level of each gene was normalized with *β*-actin mRNA content.

### 2.9. Statistical Analysis

All data are expressed as mean ± standard deviation (SD) and analyzed by one-way ANOVA or Student's *t*-test. All statistical analyses were performed using GraphPad Prism 6.0, with statistical significance at *P* < 0.05.

## 3. Results

### 3.1. GLP Attenuates Symptoms of DSS-Induced Acute Colitis

**Figure 1:**
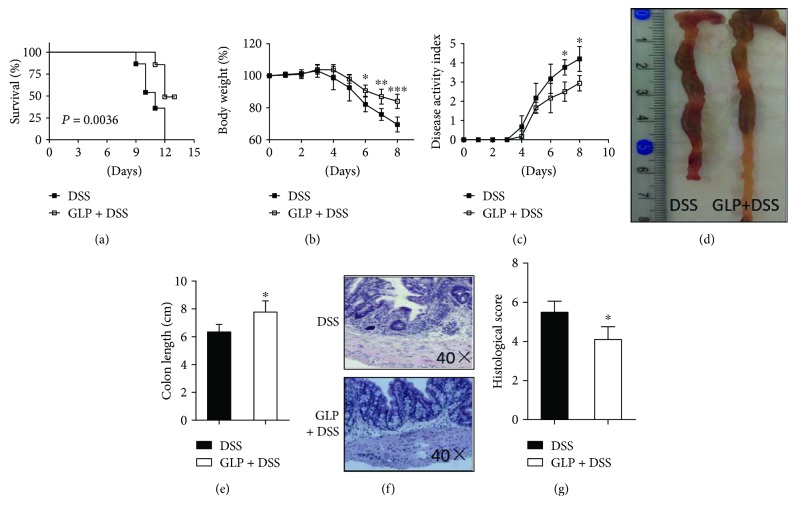
GLP attenuated DSS-induced acute colitis. Mice were orally treated with 2.5% DSS in drinking water to induce colitis as described in the Methods. On day 7 after induction of colitis, changes were observed in (a) survival rate (*n* = 10); (b) body weight (%); (c) disease activity index (DAI); (d–e) colon length; (f–g) colon histopathological damage scores. The data are presented as mean ± SD (GLP + DSS-induced acute colitis versus DSS-induced acute colitis, ^∗^*P* < 0.05; ^∗∗^*P* < 0.01; and ^∗∗∗^*P* < 0.001) (*n* = 10). DSS: dextran sulfate sodium; GLP: *Ganoderma lucidum* polysaccharides.

All mice orally administered with 2.5% DSS for 6 days showed symptoms of colitis, including diarrhea, loss of body weight, and rectal bleeding. Pretreatment with GLP prevented the development of DSS-induced acute colitis, reflecting better survival, loss of body weight, shortened colon length, histological changes of colon tissues, and DAI score. Compared with control mice, GLP-treated mice exhibited obviously enhanced survival rates (Figure 1(a)), lower loss of body weight (Figure 1(b)), decreased DAI score initially on day 7 (Figure 1(c)), less colon shortening (Figures 1(d) and 1(e)), and decreased histological scores (Figures 1(f) and 1(g)). Thus, these results suggested that GLP treatment had beneficial effects on acute colitis of DSS induced in mice.

### 3.2. Cell Accumulation in LPL of Colon in GLP-Treated Mice during DSS-Induced Acute Colitis

**Figure 2:**
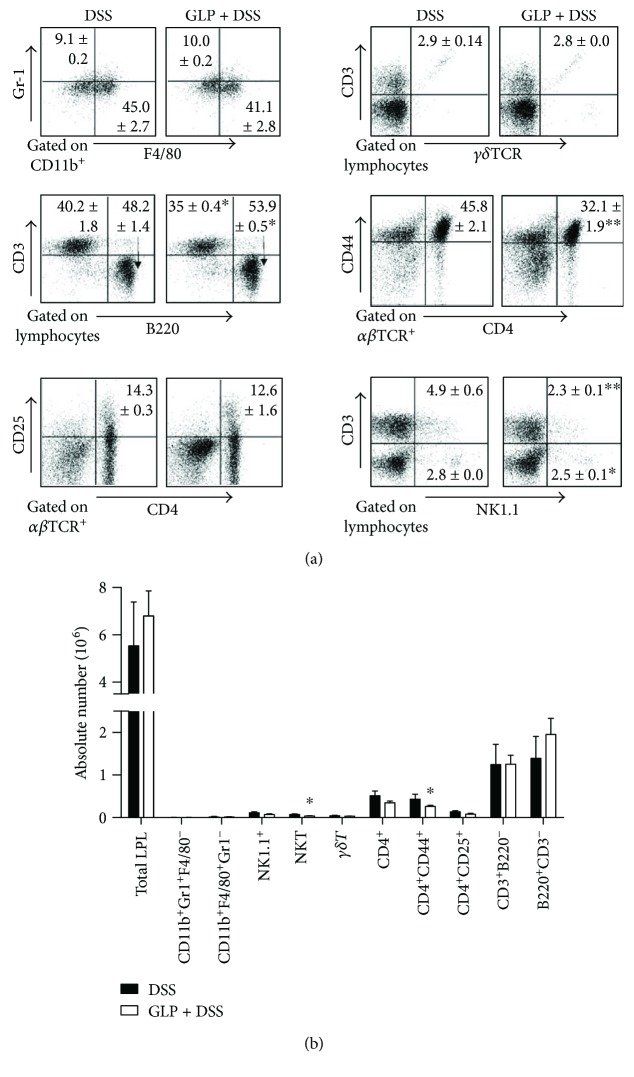
Flow cytometry analysis of the populations of LPL in the colon of mice. (a) The frequencies of neutrophils (CD11b^+^ Gr1^+^ F4/80^−^), macrophages (CD11b^+^ F4/80^+^ Gr1^−^), *γδ*T cells (*γδ*TCR^+^), NK cells (NK1.1^+^ CD3^−^), NKT cells (NK1.1^+^ CD3^+^), CD4^+^, CD4^+^ CD44^+^ (effector T cells), CD4^+^ CD25^+^ (regulatory T cells), CD3^+^ B220^−^ (T cells), and B220^+^ CD3^−^ (B cells) in the LPL of colon on day 7 after administration with DSS-only or GLP + DSS. (b) The absolute cell numbers of all kinds of cells were calculated. Data indicated mean ± SD of three mice of each group were obtained from a representative of three independent experiments (^∗^*P* < 0.05 and ^∗∗^*P* < 0.01).

No significant differences in the percentages and absolute numbers of neutrophils (CD11b^+^ Gr1^+^ F4/80^−^), macrophages (CD11b^+^ F4/80^+^ Gr1^−^), and *γδ*T cells in LPL of colons in control and GLP group mice were observed. In the GLP-treated acute colitis group, the percentage of B220^+^CD3^−^ cells in LPL significantly increased in GLP-treated mice, while CD3^+^ B220^−^ cells in LPL significantly decreased compared with control mice, but the absolute number of B220^+^CD3^−^ cells and CD3^+^ B220^−^ cells showed no differences in the two groups. The percentages and absolute numbers of CD4^+^ CD44^+^ in LPL were significantly lower in the GLP-treated acute colitis group than in the control group. The percentages and absolute numbers of CD4^+^ CD25^+^ T cells demonstrated no significant differences in the two groups. The percentage of NK cell (NK1.1^+^ CD3^−^) and NKT cells (NK1.1^+^ CD3^+^) in LPL of GLP-treated mice significantly reduced compared to untreated mice (Figure 2(a)), while the absolute numbers of only NKT cells (NK1.1^+^ CD3^+^) of LPL in the GLP-treated mice significantly decreased compared with controls (Figure 2).

**Figure 3:**
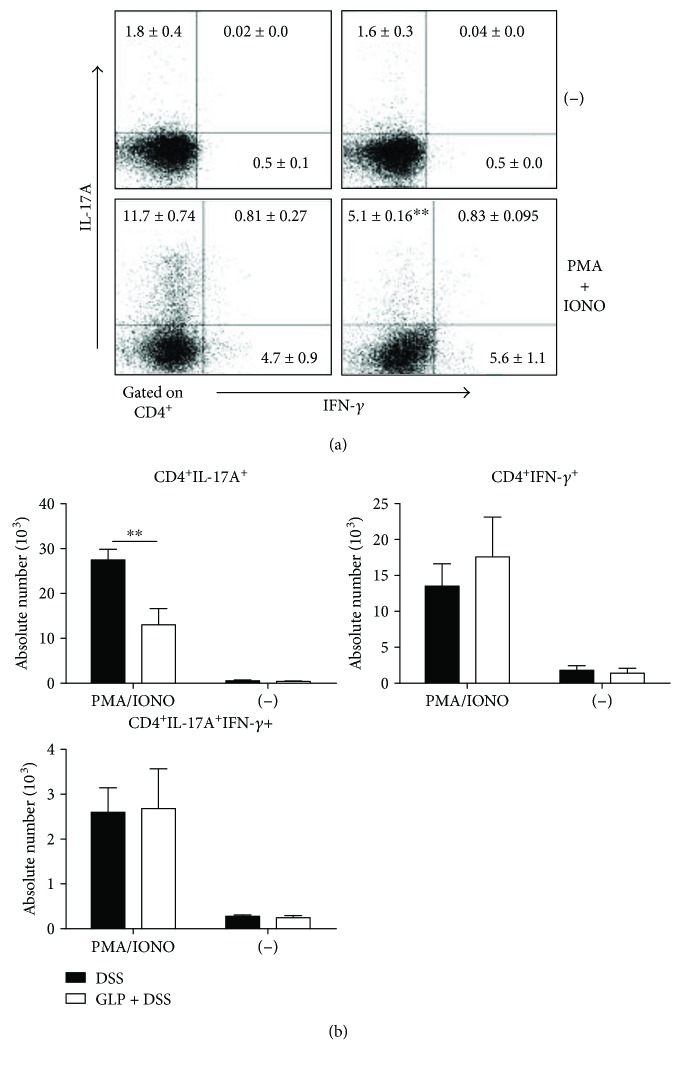
Cytokine-producing T cells in the LPL of colon in mice. (a) The frequencies of CD4^+^ IL-17A^+^, CD4^+^ IFN-*γ*^+^, and CD4^+^ IL-17A^+^ IFN-*γ*^+^ T-LPLs. (b) The absolute number of cytokines producing CD4^+^ IL-17A^+^, CD4^+^ IFN-*γ*^+^ T-LPLs, and CD4^+^ IL-17A^+^ IFN-*γ*^+^ T-LPLs with and without stimulation. Data indicated mean ± SD of three mice of each group were obtained from a representative of three independent experiments (^∗^*P* < 0.05 and ^∗∗^*P* < 0.01).

Furthermore, we detected IFN-*γ*- or IL-17A-producing CD4^+^ T cells in the lamina propria lymphocytes (T-LPLs) in GLP-treated and untreated acute colitis mice. There was a significant decrease in the percentage and absolute number of IL-17A-producing CD4^+^ T-LPLs in GLP-treated mice compared with control mice after phorbol 12-myristate 13-acetate (PMA)/ionomycin stimulations. The percentages and absolute numbers of IFN-*γ*-producing CD4^+^ T-LPLs showed no marked differences between these two groups (Figure 3). These results suggested that the effect of GLP may promote the proliferation of B cells, regulate Th1, Th17, and Th2 cell responses, and inhibit the proliferation NK cell and NKT cells in LPL of colon in DSS-induced acute colitis mice.

### 3.3. GLP Treatment Decreases Expression of Cytokines and Transcription Factor in the Intestine of Mice with DSS-Induced Acute Colitis

**Figure 4:**
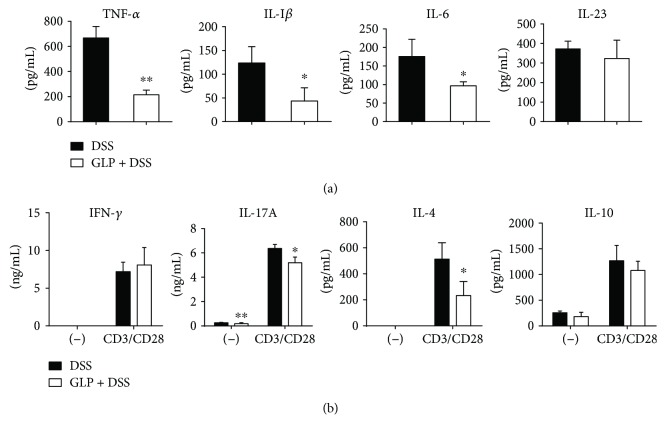
Cytokine production in LP of colon as analyzed by ELISA. (a) Unstimulated cells; (b) cells with TCR (anti-CD3 and anti-CD28 mAbs) stimulations. Data indicated mean ± SD of three mice of each group were obtained from a representative of three independent experiments (^∗^*P* < 0.05 and ^∗∗^*P* < 0.01).

**Figure 5:**
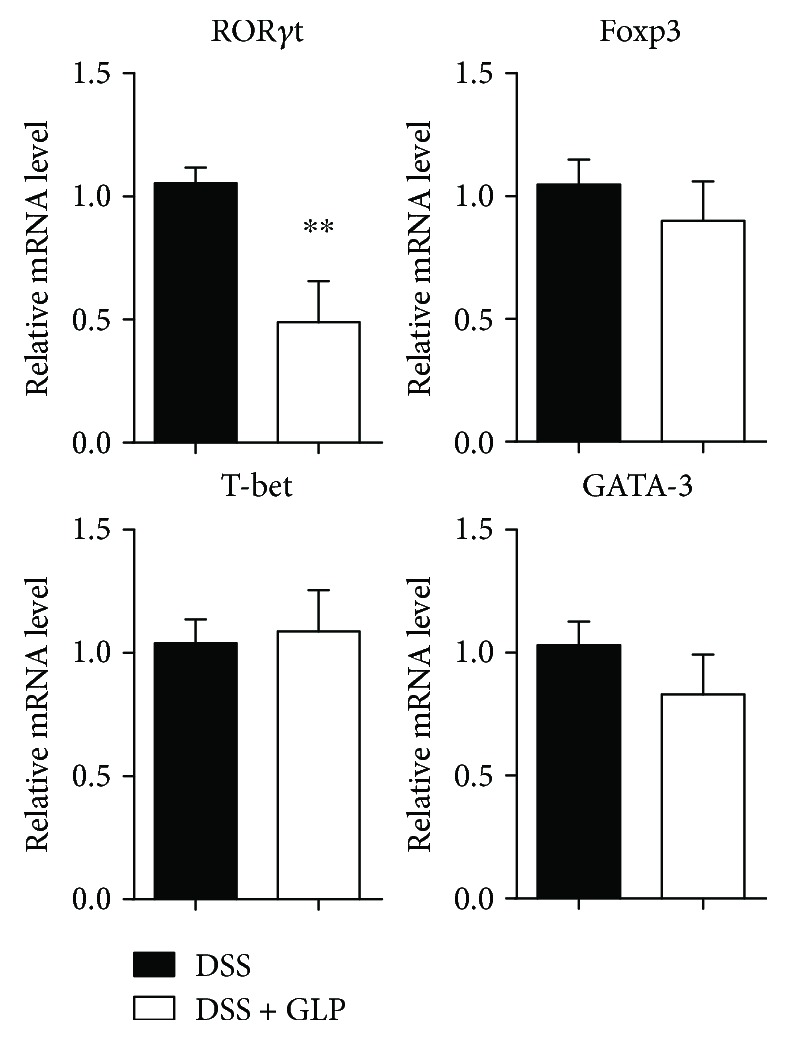
The mRNA expression of the transcription factor. Total mRNA was extracted from colonic tissues to analyze the mRNA expression of ROR-*γ*t, Foxp3, T-bet, and GATA-3 by real-time PCR. The data are presented as mean ± SD of six mice of each group obtained from a representative of three independent experiments (^∗^*P* < 0.05 and ^∗∗^*P* < 0.01).

Measurement of cytokine concentrations in the culture supernatants of unstimulated LPL manifested that the levels of TNF-*α*, IL-1*β*, and IL-6 were significantly decreased in the GLP-treated acute colitis group compared with the control group. The level of IL-23 showed no significant difference between these two groups. The level of IL-17A with anti-CD3 and anti-CD28 mAbs stimulation and without any stimulation was significantly reduced in the GLP-treated acute colitis group compared with the control group. The levels of IL-4 were significantly decreased with anti-CD3 and anti-CD28 mAbs stimulation, and similar results were observed without any stimulation in the supernatants of LPL of the GLP-treated acute colitis group compared with the control group. However, a similar concentration of IFN-*γ* and IL-10 was observed with or without anti-CD3 and anti-CD28 mAbs stimulation between these two groups (Figure 4). Also, GLP treatment led to a reduction in the mRNA expression of ROR-*γ*t. (Figure 5). However, it did not lead to any change in the mRNA expression of Foxp3, T-bet, and GATA-3 (Figure 5).

## 4. Discussion

In this study, we investigated the protection mechanism of GLP on acute colitis induced by DSS in mice. IBD is considered as Th1- or Th2-mediated inflammatory disease [2, 3]. Th17 was recently reported to play a pivotal role in the pathogenesis of DSS-induced colitis and human IBD [14, 43]. GLP has strong immunomodulatory activity, especially on innate and adaptive immune responses [29, 44]. However, the ability of GLP to suppress DSS-induced acute colitis by its immunomodulatory effect has not been confirmed.

Our results proved the beneficial effects of GLP on the symptoms of colitis, which were depicted when assessing the survival rate, body weight loss, colon length, histopathological scores, and DAI score (Figure 1). Recent results showed that polysaccharides derived from *G. lucidum* fungus mycelia ameliorate indomethacin-induced small intestinal injury by induction of granulocyte macrophage colony-stimulating factor (GM-CSF) from macrophages [45]. However, the result of our study showed that GLP had no effects on the populations of macrophage and the neutrophils in colonic LP in mice with DSS-induced acute colitis (Figure 2).

The protective effects of GLP on colitis induced by DSS were associated with an increase in B cells but a decrease in T cells, mainly a decrease in 17A-producing CD4^+^ T-LPLs, and a decrease in the production of IL-17A and IL-4 in LP (Figures 2, 3, and 4). Recently, it has been reported that a novel subset of helper CD4^+^ T cells producing IL-17A, namely, Th17 cells, was involved in the progression of DSS-induced colitis [46]. Also, we showed that the mRNA expression levels of ROR-*γ*t, which is the principal regulator for Th17 differentiation [47], decreased in the colon tissue of GLP treated mice (Figure 5). IL-23 has been shown to be an important modifier of Th17 cell phenotype in the intestine [48]. However, GLP administration showed no improvement in the production of IL-23 in the colon of DSS mice, indicating that the administration of GLP probably alleviated the extent of acute intestinal inflammation via downregulation of Th17 cell responses independent of IL-23. Otherwise, it has been reported that GLP produce immune responses with an overall Th1 bias, with high levels of IFN-*γ* and IL-2, as well as IL-10 and IL-17 [49, 50], but there are still different views regarding the effect of GLP on IL-4 [34, 49, 50]. These results suggested that GLP exerted immunomodulating responses on colitis possibly through altering the balance of Th1/Th2 and Th17/Treg. The mechanism needs further investigation. In addition, recent study results showed that the level of SIgA from B cells in the intestine was significantly increased in the ileum of rats with oral administration of GLP and was commonly considered as a critical effector molecule to protect the mucosal surfaces [34].

NK T cells exert an effector function, for instance, expression of IL-17 and IL-22 in the intestine [51]. We showed that the percentages and absolute numbers of NKT cells were significantly reduced in the colonic LP of GLP-treated mice (Figure 2). It may play a significant role in the production of IL-17 by NKT cells in the induction of IBD, as increased expression of IL-17 has been reported in both animal IBD models [52, 53] and patients with IBD [54, 55]. Our data demonstrated that the percentage of NK cells was markedly decreased in the colonic LP of mice with GLP administration. NK cells produce cytokines, including TNF-*α* and IFN-*γ* [56], and probably might be a reason for the decrease in TNF-*α*, which has a key role in IBD. Antibodies against TNF-*α* were successfully accomplished in clinical applications [57].

Cytokines play a critical role in the development and progression of inflammatory responses. To assess whether the positive effects of GLP on colitis were involved in the deceased secretion of proinflammatory cytokines, expression levels of TNF-*α*, IL-1*β*, and IL-6 in the colonic LP in colitis mice treated with or without GLP were measured. Out results demonstrated that the levels of TNF-*α*, IL-1*β*, and IL-6 were markedly downregulated in the colonic LP in colitis mice with GLP treatment (Figure 4). These results were consistent with the findings of the previous report, which showed that the GLBR, an extract of *G. lucidum* grown on brown rice, inhibited the expression of TNF-*α*, IL-1*β*, IL-6, and COX-2 by inhibition of MAPK and NF-*κ*B pathways in mice with DSS induced colitis [26]. NLRP3 inflammasome is a multimolecular cytosol complex that, when activated, leads to the cleavage of pro-IL-1*β* to IL-1*β*. In an acute colitis mouse model induced by DSS, NLRP3 gene knockout and medical inhibition of the NLRP3 inflammasome activation both exerted protective effects on mice [58, 59]. Therefore, GLP had an anti-inflammatory effect on colonic LP in colitis mice, possibly due to the decreasing production of proinflammatory cytokines and inhibiting NLRP3 inflammasome signaling. The next study needs to confirm it.

In summary, we herein described that polysaccharides isolated from *G. lucidum* GLP may ameliorate acute colitis through the suppression of the immune responses, including decreased secretion of proinflammatory cytokines, such as TNF-*α*, IL-6, IL-1*β*, and IL-17A, increased the populations of B cells and decreased the populations of Th17 cells, NK cells, and NKT cells. There are various pharmaceutical IBD treatment options available, while they have their own limitations of both efficacy and safety [60]. Hence, our results suggest that polysaccharides with valuable effects may be used as a novel therapeutic approach to mitigate acute inflammation in IBD individuals. Moreover, detailed research studies including clinical trials are warranted to verify the potential of these promising immune modulatory agents.

## Figures and Tables

**Table 1:** Primer sequences for real-time PCR.

Gene	Forward	Reverse
T-bet	CCAGGGAACCGCTTATATGT	CTGGGTCACATTGTTGGAAG
GATA-3	ACAGCTCTGGACTCTTCCCA	GTTCACACACTCCCTGCCTT
ROR-*γ*t	CCACTGCATTCCCAGTTTCT	CGTAGAAGGTCCTCCAGTCG
Foxp3	GGCCCTTCTCCAGGACAGA	GCTGATCATGGCTGGGTTGT
*β*-Actin	TTCCAGCGTTCCTTCTTGGGT	GTTGGCATAGAGGTGTTTACG
